# Psychiatric hospitalizations by the Unified Health System in Brazil between 2000 and 2014

**DOI:** 10.11606/s1518-8787.2021055002155

**Published:** 2021-04-07

**Authors:** Hugo André da Rocha, Ilka Afonso Reis, Marcos Antônio da Cunha Santos, Ana Paula Souto Melo, Mariangela Leal Cherchiglia

**Affiliations:** I Universidade Federal de Minas Gerais Faculdade de Medicina Programa de Pós-Graduação em Saúde Pública Belo HorizonteMG Brasil Universidade Federal de Minas Gerais. Faculdade de Medicina. Programa de Pós-Graduação em Saúde Pública. Belo Horizonte, MG, Brasil; II Universidade Federal de Minas Gerais Instituto de Ciências Exatas Departamento de Estatística Belo HorizonteMG Brasil Universidade Federal de Minas Gerais. Instituto de Ciências Exatas. Departamento de Estatística. Belo Horizonte, MG, Brasil; III Universidade Federal de São João Del Rei Faculdade de Medicina DivinópolisMG Brasil Universidade Federal de São João Del Rei. Faculdade de Medicina. Divinópolis, MG, Brasil; IV Universidade Federal de Minas Gerais Faculdade de Medicina Departamento de Medicina Preventiva e Social Belo HorizonteMG Brasil Universidade Federal de Minas Gerais. Faculdade de Medicina. Departamento de Medicina Preventiva e Social. Belo Horizonte, MG, Brasil

**Keywords:** Mental Disorders, Day Care, Medical, Hospitalization, Commitment of Mentally Ill, Unified Health System, Mental Health, Public Health Policy

## Abstract

**OBJECTIVE:**

To characterize the profile of patients hospitalized for mental and behavioral disorders by the Unified Health System (SUS) in Brazil between 2000 and 2014, and to verify how aspects of the new mental health policy influenced the rate of hospitalized patients in that period.

**METHODS:**

Non-concurrent prospective cohort study using secondary data from inpatients with a primary diagnosis of mental and behavioral disorders between 01/01/2000 and 12/31/2014. Sociodemographic, clinical, and hospital characteristics variables were selected. Overall rates of hospitalized patients were calculated according to reason for admission, type of hospital, legal nature, and number of admissions per year for each patient. The association between rates of hospitalized patients, number of psychiatric beds per year, and number of Psychosocial Care Centers per year were tested.

**RESULTS:**

We selected a total of 1,549,298 patients, whose most frequent diagnoses on first admission were psychoactive substance use disorders, followed by schizophrenia and mood disorders. The median of hospitalizations per patient was 1.9 and the length of stay per patient was 29 days. The overall rate of hospitalized patients was reduced by almost half in the period. The number of beds per year was positively associated with the rates of hospitalized patients; the number of CAPS per year was negatively associated with some rates of hospitalized patients.

**CONCLUSION:**

Even in the face of adversity, the National Mental Health Policy has advanced in its goal of progressively reducing hospital beds and increasing the supply of substitute services such that both strategies were associated with the reduced inpatient rates. But the changes were felt with greater intensity in the first years of the policy’s implementation, becoming less pronounced in recent years.

## INTRODUCTION

The National Mental Health Policy (PNSM) underwent significant transitions in recent years. Published on April 6, 2001, Law 10,216 defined the legal framework for ensuring the rights of people with mental disorders, creating conditions for them to be treated, preferably, in community mental health services. From the moment the law came into force, hospitalization, whatever its modality, would only be assigned when out-of-hospital resources proved insufficient; to do so, the country had to invest in the creation of out-of-hospital services capable of meeting the demand and providing due treatment^a^.

Psychosocial Care Centers (CAPS) are community mental health services that operate according to the logic of territories. Understood as strategic devices of the policy, they are structures that primarily serve people with severe or persistent mental disorders, and individuals with disorders caused by alcohol and drug use. Established in 2002, they were defined according to population size and target audience (CAPS I, CAPS II, CAPS III, CAPS alcohol and drugs (AD) and Child and Youth (CAPS i))^b^. After a period of great expansion in the implementation of new services, we have seen a slowdown in the process in recent years. From 2002 to 2007, we had an average 25.9% annual growth of new CAPS; in the following period, from 2008 to 2014, the average annual increase was 9.8%^c^.

Alongside the structuring of new devices, we had the progressive reduction of hospital beds, which occurred in a heterogeneous manner in the different regions of the country^1^. The National Program for Evaluating the Hospital System/Psychiatry (PNASH/Psiquiatria)^d^ and the Annual Program for Restructuring Psychiatric Hospital Care in the SUS (PRH)^e^ played a key role in reorganizing the supply of hospital beds. Moreover, the slow progress in implementing full-time services (CAPS III and CAPS AD III), capable of ensuring back-up for crises episodes, hindered reducing hospital admissions^1^.

Previous studies undertook the challenge of assessing changes in hospitalization rates using data provided by Datasus with municipal or state scope^4^. A limitation imposed on these studies is the non-identification of patients in the available data, making it impossible to indicate the course of hospitalizations and individualized analysis by patient. We must also highlight the importance of investigating the role of strategies implemented under PNSM in reducing psychiatric hospitalizations.

Based on the above, we present two research questions: 1) what is the profile of patients hospitalized for mental disorders; and 2) what is the influence of the strategies implemented on the behavior of rates of hospitalized patients?

In this sense, this study aims to characterize the profile of patients hospitalized for mental and/or behavioral disorders by the Unified Health System (SUS) in Brazil between 2000 and 2014, and to verify how aspects of the PNSM influenced the rate of hospitalized patients in that period. The investigation will use integrated data via deterministic-probabilistic linkage, in which we were able to individualize the admissions of each patient

## METHODS

This is a non-concurrent prospective cohort study using secondary data from patients selected from the National Database of Health, an individual-centered database built with record linkage techniques integrating data from the main information systems of the Unified Health System: Outpatient Information System (SIA), Hospital Information System (SIH) and Mortality Information System (SIM), from 2000 to 2015^14^.

As inclusion criteria, we selected all patients registered in the SIH whose primary diagnosis referred to a code on Chapter V of the International Statistical Classification of Diseases and Related Health Problems, 10th Revision – ICD 10 Mental and behavioral disorders (F00-F99), and whose date of hospitalization was between 01/01/2000 and 31/12/2014. Records of patients under 18 years old at the time of admission were removed.

To describe the profile of patients and hospitalizations, we selected the following variables: I – sociodemographic: gender (female, male), age (in years) and region of residence (North, Northeast, Southeast, South, Midwest); II – clinical: reason for hospitalization (primary diagnosis), occurrence of death in the period (no, yes), occurrence of death during psychiatric hospitalization (no, yes), length of stay in days (sum of the hospitalized days of each patient) and number of hospitalizations per patient; III - characteristics of the facility: type of facility (specialized hospital, general hospital, others) and legal nature of the facility (public, private, private non-profit).

Psychiatric hospitalizations are characterized by long stays, being recommended that consecutive authorization for hospital admittance (AIH) keep the number of the initial AIH, but new records are often generated, practice that ends up masking the long duration of psychiatric hospitalization. To mitigate this effect, as previously done by other authors^12^, we verified the AIHs of each patient, considering it a single hospitalization when the subsequent AIH took place in the same hospital as the previous one and: a) the admission date of the subsequent AIH was the same as the discharge date of the previous AIH or; b) the admission date of the subsequent AIH had one day different from the discharge date of the previous AIH or; c) the admission date of the subsequent AIH was contained within the hospitalization period of the previous AIH.

For the descriptive analysis of the continuous variables, we used the median and the interquartile range (IQR), since data distribution was not symmetrical. Categorical data were reported as frequency and percentage.

We built time series listing the patients hospitalized in each of the years under study (2000 to 2014); to calculate the rates of hospitalized patients we considered the patient’s first admission in each year, disregarding the possible readmissions. Hospitalizations that exceeded the calendar year were computed in the year in which they began. The rates of hospitalized patients for each year were calculated considering the following equation:

$$\frac{\text{ number of patients hospitalized in the year }}{\text{ total population in the year }(\geq 18\text{ years old })}\times 100.000\text{ inhabitants }$$

For the years 2000 and 2010, we took as reference the population of census^15^, and for the others, we considered the population projections made by the Brazilian Institute of Geography and Statistics^16^. We standardized the rates of hospitalized patients per year by gender and age using the direct method, having as standard the Brazilian population according to the 2010 census^15^. Besides the general rate of hospitalized patients, we also calculated specific rates according to: reason for hospitalization, type of hospital, legal nature of the hospital and number of hospitalizations by patient per year.

Time series regression was used to verify whether the variation identified over the period was associated with time. In this regression model, the response variable is the time series itself and time is the explanatory variable of the model, so that the regression equation explains the variation of the inpatient rate as a function of time^17^. Each of the standardized rates of hospitalized patients was tested individually with the variable indicating the time in years. Values of p < 0.05 were considered statistically significant. The Durbin-Watson test was used to verify the occurrence of autocorrelation. Rates of hospitalized patients who presented significant variation were selected for the next modeling stage.

Considering that some strategies were implemented under PNSM during the study period, we verified whether two of these actions were associated with changes in the rates of hospitalized patients. These strategies analyzed were: the reduction of psychiatric beds and the expansion of CAPS. For this analysis, we considered the total number of beds available per year and the total number of CAPS per year (excluding CAPS i, focused on the care of children and adolescents, not included in this study).

All values referring to the CAPS variables and the total number of beds available per year were taken from the official reports published by the Ministry of Health^c,f^.

To test the association between the rates of hospitalized patients and the explanatory variables number of beds and number of CAPS per year, we used generalized least squares (GLS) model with first-order auto-regression process. Since the explanatory variables are also configured as time series, we had to use a regression model capable of incorporating autocorrelation into the error covariance structure. For each rate of hospitalized patients, 2 regression models were evaluated, one considering the beds per year and the other the CAPS per year. These two variables are highly correlated, making any regression model with both included unfeasible. The quality of the model fit was verified by graphical analysis of the residues and the statistical analyses were conducted in the R Project for Statistical Computing (version 3.6.1) programming.

This study is part of the project “Epidemiological, economic and care trajectories evaluation of high-cost procedures in SUS: use of a patient-centered database based on the integration of health information systems records,” approved by UFMG’s Research Ethics Committee – (CAAE 44121315.2.0000.5149).

## RESULTS

We selected a total of 1,549,298 patients, most of them male. The region of the country with the highest number of hospitalized patients was the Southeast, and the region with the lowest number the North. Psychoactive substance use disorders, followed by schizophrenia and mood disorders were the most frequent reasons for hospitalization. We verified occurrence of death for 12.2% of the patients, of which 7.7% died during a psychiatric hospitalization (Table 1).

**Table 1:** Characteristics of patients hospitalized for mental and/or behavioral disorders by SUS and hospitals, Brazil, 2000–2014.

Patients	n	(%)
	1.549.298	(100)
Gender		
Female	550,464	35.5
Male	998,834	64.5
Region		
North	33,651	2.2
Northeast	303,886	19.6
Southeast	673,688	43.5
South	403,229	26.0
Midwest	134,844	8.7
Reason for Hospitalization by ICD-10 Group		
F10-F19 Mental and behavioral disorders due to psychoactive substance use	609,822	39.4
F20-F29 Schizophrenia, schizotypal and delusional disorders	532,083	34.3
F30-F39 Mood [affective] disorders	238,164	15.4
F00-F09 Organic, including symptomatic, mental disorders	88,569	5.7
F40-F99 Other Mental Disorders	80,660	5.2
Death		
No	1,35,528	87.8
Yes	189,770	12.2
Death during psychiatric hospitalization		
No	175,207	92.3
Yes	14,563	7.7
Number of hospitalizations per patient		
One	1,043,048	67.3
Two	240,210	15.5
Three	162,212	10.5
Four or more	103,828	6.7
Type of hospital		
Specialized	1,020,276	65.9
General	461,943	29.8
Others	35,197	2.2
N.A.*	31,882	2.1
Legal nature of the hospital		
Non-profit private	551,526	35.6
Private	511,378	33.0
Public	483,711	31.2
N.A.*	2,683	0.2

* Facilities that had records only for periods prior to August 2005 could not be classified due to the time limitation of the CNES data.

Most patients had a single admission over the period. Hospitalizations were more frequent in specialized hospitals and, regarding the legal nature, the highest proportion of hospitalizations occurred in private non-profit hospitals.

Patients’ median age was 38 years (interquartile range [IQR] 29:47). The median number of hospitalizations for each patient was 1.9 times during the entire period studied (IQR 1–2), and the median length of stay per patient was 29 days (IQR 9–62). We found a total of 2,957,767 hospitalizations in the period studied (data not presented in table).

**Figure 1:**
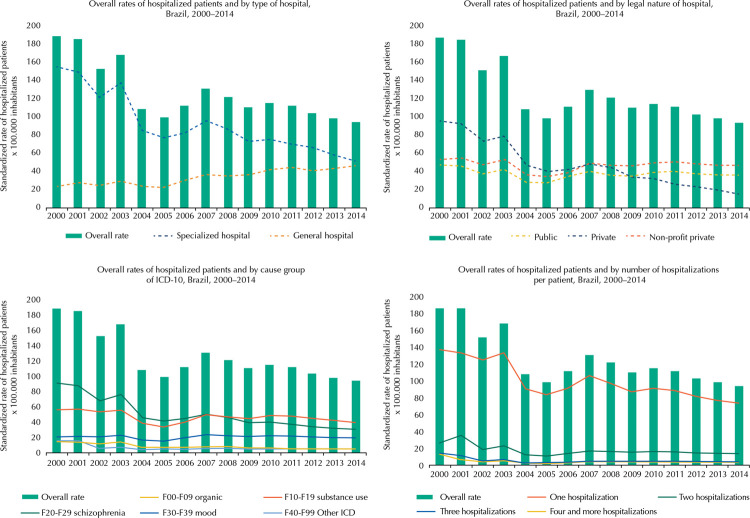
General and specific rates of hospitalized patients, Brazil.

Figure 1 shows the rates of hospitalized patients standardized by age and gender. The overall rate of hospitalized patients showed a downward trend over the period, going from 188.5 in the first year to 94.4 per 100,000 inhabitants in the last year, representing a 49.9% reduction. Regarding the rate of hospitalized patients per type of hospital, we observed a reduction in hospitalizations in specialized hospitals (from 154.4 to 51.2) and a simultaneous increase in general hospitals (from 24 to 46). As for the rate by legal nature of the hospital, we highlight a 84.32% reduction of patients admitted to private hospitals, from 95.7 in 2000 to 15 per 100,000 inhabitants in 2014.

* Rates of hospitalized patients standardized by gender and age, 2000–2014.

The rate of hospitalized patients by diagnostic group indicates a 66% reduction in the rates of patients hospitalized for schizophrenia (from 91.5 to 30.5), while the rate for psychoactive substance use showed a 29.8% reduction (from 56.6 to 39.7). On the number of hospitalizations per patient, the rate of patients submitted to a single hospitalization per year showed the same downward trend of the overall rate, from 137.3 in 2000 to 74 per 100,000 inhabitants in 2014.

Times series regression revealed a significant variation in the rates of hospitalized patients as a function of time. Excepting the rate of patients admitted to general hospitals, which showed an increasing trend, all other significant rates indicated a decreasing relationship, i.e., a tendency to decrease over time. The overall rate of hospitalized patients showed a reduction of 5.72 hospitalized patients per 100,000 inhabitants each year. The coefficients for each of the models can be seen in Table 2.

**Table 2:** Time series regression coefficients according to general and specific rates of hospitalized patients, Brazil, 2000–2014.

Standardized rate of hospitalized patients per year	Intercept (95%CI)^a^	Time (95%CI)^a^	Adjusted R^2^	p	Tendency
Overall rate of hospitalized patients	172.61 (149.82 to 195.39)	-5.72 (-8.22 to -3.21)	0.62	< 0.001	Reduction
Rate by cause group					
F00-F09 organic disorders	14.22 (12.15 to 16.27)	-0.7 (-0.93 to -0.48)	0.76	< 0.001	Reduction
F10-F19 substance use	53.28 (45.77 to 60.78)	-0.8 (-1.62 to 0.02)	0.19	0.06	^b^
F20-F29 schizophrenia	82.87 (71.60 to 94.13)	-3.96 (-5.19 to -2.72)	0.77	< 0.001	Reduction
F30-F39 mood disorders	20.57 (17.82 to 23.31)	0.03 (-0.27 to 0.32)	-0.07	0.85	^b^
F40 to F99 other ICDs	11.17 (7.49 to 14.84)	-0.54 (-0.94 to -0.13)	0.34	< 0.05	Reduction
Rate by type of hospital					
Specialized hospital	144.82 (127.19 to 162.43)	-6.6 (-8.53 to -4.66)	0.79	< 0.001	Reduction
General Hospital	19.93 (15.86 to 24.01)	1.7 (1.25 to 2.14)	0.83	< 0.001	Increase
Rate by legal nature of the hospital					
Public Hospital	40.11 (33.69 to 46.53)	-0.32 (-1.02 to 0.38)	-0.003	0.35	^b^
Private Hospital	91.14 (80.15 to 102.12)	-5.45 (-6.65 to -4.23)	0.87	< 0.001	Reduction
Non-profit Private Hospital	47.72 (40.54 to 54.90)	-0.09 (-0.88 to 0.69)	-0.07	0.80	^b^
Rate by number of hospitalizations per year for each patient					
One hospitalization per year	133.40 (119.60 to 147.19)	-4.16 (-5.67 to -2.64)	0.71	< 0.001	Reduction
Two hospitalizations per year	24.93 (18.83 to 31.02)	-0.9 (-1.56 to -0.22)	0.34	< 0.05	Reduction
Three hospitalizations per year	8.84 (5.95 to 11.73)	-0.43 (-0.74 to -0.10)	0.35	< 0.05	Reduction
Four or more hospitalizations per year	6.86 (4.21 to 9.51)	-0.31 (-0.59 to -0.01)	0.23	< 0.05	Reduction

^a^ 95%CI: 95% confidence interval.

^b^ showed no statistical significance.

Generalized least square models indicated an association of the variable number of beds per year for almost all rates of hospitalized patients, with almost all models showing positive coefficients. As the coefficients show low values, we had to adopt a multiplication factor to simplify the interpretation. Thus, for the rate of patients admitted to specialized hospitals, for example, the increase of 1000 beds would result in an increase of 3 hospitalized patients per 100,000 inhabitants/year. The number of CAPS per year was significant in fewer models, with negative coefficients. Considering also the rate of patients admitted to specialized hospitals, we observed that an increase of 1000 CAPS units would result in a reduction of 49 hospitalized patients per 100,000 inhabitants/year. All coefficients and their confidence intervals (95%) can be seen in Table 3.

**Table 3:** Coefficients of generalized least square regressions according to general and specific rates of hospitalized patients by number of CAPS per year and number of psychiatric beds per year, Brazil, 2000–2014.

Standardized rate of hospitalized patients per year	CAPS		Beds	
	Coefficient (95%CI)^a^	p	Coefficient (95%CI)^a^	p
Overall rate of hospitalized patients	-0.043 (-0.09 to 0.004)	0.075	0.003 (0.001 to 0.004)^b^	< 0.001
Rate by cause group				
F00-F09 organic disorders	-0.005 (-0.008 to -0.002)	0.005	0.0003 (0.0002 to 0.0004)	< 0.001
F20-F29 schizophrenia	-0.024 (-0.067 to 0.018)	0.242	0.002 (0.001 to 0.002)^b^	0.000
F40 to F99 other ICDs	-0.004 (-0.017 to 0.008)	0.479	0.0003 (0.0000008 to 0.001)^b^	0.049
Rate by type of hospital				
Specialized hospital	-0.049 (-0.083 to -0.016)	0.007	0.003 (0.002 to 0.003)^b^	< 0.001
General Hospital	0.013 (0.009 to 0.017)	0.000	-0.001 (-0.001 to -0.00008)	0.025
Rate by legal nature of the hospital				
Private Hospital	-0.038 (-0.07 to -0.007)	0.020	0.002 (0.002 to 0.003)	< 0.001
Rate by number of hospitalizations per year for each patient				
One hospitalization per year	-0.03 (-0.053 to -0.008)	0.013	0.002 (0.001 to 0.003)^b^	< 0.001
Two hospitalizations per year	-0.006 (-0.015 to 0.002)	0.141	0.0004 (0.00005 to 0.0007)^b^	0.025
Four or more hospitalizations per year	-0.003 (-0.011 to 0.005)	0.497	0.0004 (0.0001 to 0.0007)^b^	0.009

^a^ 95%CI: 95% confidence interval.

^b^ Non-significant intercept (5% significance level).

## DISCUSSION

The results presented, using a nationwide non-concurrent prospective cohort, indicate the existence of changes in mental disorder hospitalizations from 2000 to 2014. We observed a reduction in both absolute values and the rates of hospitalized patients, as well as changes in the profile of services, with increased hospitalizations in general hospitals and reduced admissions in specialized hospitals. It should be noted that this study uses as main indicator the rate of hospitalized patients (per 100,000 inhabitants), which differs from the rate of hospitalizations (per 100,000 inhabitants). This allowed us to evaluate the reduction in the number of individuals hospitalized each year.

We found that most of the population analyzed are male, which corroborates other studies that also evaluated patient hospitalization for mental disorders^5,7,11^. A study conducted in the Southern region of Brazil identified a higher percentage of women hospitalized in relation to men; but it is relevant to consider that this research did not include patients hospitalized for alcohol and other drugs^18^. Regarding age, we observe that these are people of working age; the age distribution shows that 75% of the patients were between 18 and 47 years old. It is uncommon to find studies with patients of advanced average age, with the highest frequency of patients being between 30 and 49 years old^5,7,11,18^.

Other studies that have investigated the time trend of psychiatric hospitalizations also found results that reinforce the hypothesis of time-associated reduction of rates^9^. The state of Rio Grande do Sul showed an increase in hospitalization rates over time^8^.

The changes in the rates of hospitalized patients by type of hospital and by legal nature of the hospital may be related to changes in hospital policy. The reduction in the rate of patients admitted to specialized hospitals was concomitant to the increase in the rate of patients admitted to general hospitals, corroborating previous studies^4^.

Psychiatric hospitalization in general hospitals may favor reducing the stigma, besides increasing access, improving care in relation to physical health and enabling consultation-liaison between the various medical specialties present in this type of hospital^4,19,20^. Data from the Global Health Observatory show that Brazil has a deficit regarding the number of psychiatric beds in general hospitals. While the country had 0.6 beds/100,000 inhabitants in 2016, countries such as Australia, Spain, Portugal and Italy had 21.8, 14.3, 11.8 and 9.0 beds/100.00 inhabitants, respectively. Neighboring countries in South America, such as Uruguay, Chile and Argentina, had 8.2, 5.2, and 2.7 beds/100,000 inhabitants, respectively^21^.

The large reduction in the rate of patients admitted to private hospitals signals changes in hiring psychiatric beds. During the period, many beds and even entire hospitals were de-accredited, as found in a study conducted with data from the state of Minas Gerais from 2001 to 2013^12^. A study published in 2007 showed that from 1995 to 2005 hospitals reduced the number of psychiatric beds by 41% (5.4 to 3.2 per 10,000 inhabitants)^2^. Between 2008 and 2017, we had a 33.3% reduction in the number of psychiatric beds in the country^1^.

We observed an important reduction in the rate of patients hospitalized for schizophrenia, to the point where the rate of hospitalized patients due to substance use has surpassed it. Previous studies have found an increase in hospitalization rates for substance use disorders; some of these studies also observed a reduction in the rate of hospitalizations for schizophrenia^8^.

The incipient structuring of non-hospital services for the care of patients diagnosed with psychoactive substance use disorders could explain the increasing rate of hospitalized patients^7^. CAPS-ad III, which treats this profile of patients on a 24-hour basis, still had low coverage during the period of this study; in 2014, there were 69 qualified services in 51 municipalities. We must also mention the increase in illicit drug use by the Brazilian population^23,24^ that every day produces a larger contingent of people who use hospitalization to treat abusive use^22^.

The rates of patients admitted once or twice a year showed less reduction than the rates of patients hospitalized three and four or more times a year. The high frequency of hospitalizations for the same patient can be understood under the revolving door phenomenon, in which patients present several hospitalizations with short discharge intervals between them. A systematic review found that a diagnosis of schizophrenia presented a higher risk of readmission^25^. In this sense, the reduced the rate of patients who were hospitalized more often may be associated with the large contraction found in the rate of patients hospitalized for schizophrenia.

Time series regression showed that most rates of hospitalized patients varied as a function of time in almost all cases, reducing each year. When evaluating the association of these changes with the strategies to reduce hospital beds and increase the number of CAPS, we observed that these isolated variables have a small effect on the variation of rates of hospitalized patients. Increasing the number of beds would imply increasing the number of hospitalized patients and, inversely, increasing the number of CAPS would reduce the number of hospitalized patients.

Previous studies have investigated CAPS relationship with admission and readmission rates, but the findings are not homogeneous. A study conducted in two Southeast metropolitan regions showed association between the increase in the coverage of CAPS services and a reduction in hospitalization rates for mental disorders^26^. Another study, conducted in the municipalities of Campinas, São Paulo, Porto Alegre and Fortaleza, found that less than 10% of users treated in CAPS required hospitalization in a period of 3 years^27^. A cohort study observed significant reductions in the occurrence of seizures, medication use, and number of psychiatric hospitalizations among users with longer time in CAPS and in intensive care^28^. In contrast to these results, a study conducted in two public psychiatric hospitals in Belo Horizonte – MG found no protective effect between CAPS coverage and the occurrence of psychiatric readmission^29^.

We can assume that introducing the CAPS has given rise to a restrained demand for mental health services, which, as previously discussed, were inaccessible, and as they become available, they inflate demand. In this sense, it is also worth mentioning that, in recent years, there has been a great increase in the coverage of primary health care teams in the country, which have incorporated actions aimed at people with mental disorders in their measures. Primary health care teams have integrated many mental health actions, especially the provision of group care and educational and health promotion actions^30^. As access and, consequently, diagnosis increase, the pressure for medium and high complexity services to meet these cases also increases.

Despite the importance of mental disorders in the global burden of disease, accounting for 9.5% of the total disability-adjusted life years lost in Brazil in 2015^24^, the investment needed to address these conditions is far from adequate. A key point of the reform, which would be the expansion of out-of-hospital services to meet the planned decrease in hospital beds, conflicts with the budgetary limit of the mental health policy^19,20^.

This study presents some limitations, such as the low number of sociodemographic variables from patients. Since it uses database information from administrative system, information such as race/color, schooling and income are often missing. Another issue was the lower volume of hospitalized patient data between 2004 and 2006, which is a limitation of the National Database of Health.

This study allowed us to expand the knowledge about the profile of patients hospitalized for mental disorders nationwide by having a comprehensive and patient-centered database. The protagonism of the psychiatric hospital in the Mental Health Policy was being diluted to other services, but without breaking with the model that for many years sustained the huge hospital park that existed in Brazil. To do so, the investments for extending extra-hospital services would need to be expanded, not the other way around, as has been observed recently. The new and severe demands in mental health undermine the ability of health managers and public administrators to meet the needs for which the worst medicine is austerity.

## Footnotes

a Brazil. Law no. 10,216, of April 6, 2001. Provides on the protection and rights of people with mental disorders and redirects the mental healthcare model. *Diário Oficial da União.* Apr. 9. 2001.

b Brazil. Ordinance/GM No. 336 of February 19, 2002. Establishes CAPS I, CAPS II, CAPS III, CAPS I II and CAPS ad II. Diário Oficial da União. Feb. 20. 2020.

c Ministry of Health. Saúde Mental em Dados – 12. Saúde Mental em Dados. 2015;10(12).

d Brazil. Ordinance no. 251, of January 31, 2002. Establishes guidelines and standards for psychiatric hospital care, reclassifies psychiatric hospitals, defines and structure the gateway for psychiatric hospitalizations in the SUS network and make other provisions. Diário Oficial da União. Feb. 1. 2002.

e Brazil. Ordinance no. 52, of January 20, 2004. Establishes the Annual Program for restructuring Hospital Psychiatric Care in the SUS. Diário Oficial da União. Jan. 20. 2004.

f Ministry of Health. Saúde Mental em Dados – 8. Saúde Mental em Dados. 2011;6(8).

## References

[1] 1. Fernandes CJ, Lima AF, Oliveira PRS, Santos WS. Índice de Cobertura Assistencial da Rede de Atenção Psicossocial (iRAPS) como ferramenta de análise crítica da reforma psiquiátrica brasileira. Cad Saude Publica. 2020;36(4):e00049519. 10.1590/0102-311x00049519 32321073

[2] 2. Andreoli SB, Almeida-Filho N, Martin D, Mateus MDML, Mari JJ. Is psychiatric reform a strategy for reducing the mental health budget? The case of Brazil. Rev Bras Psiquiatr. 2007;29(1):43-6. 10.1590/S1516-44462006005000032 17435928

[3] 3. Pitta AMF. Um balanço da reforma psiquiátrica brasileira: instituições, atores e políticas. Cienc Saude Colet. 2011;16(12):4579-89. 10.1590/S1413-81232011001300002 22124894

[4] 4. Candiago R, Abreu PB. Uso do Datasus para avaliação dos padrões das internações psiquiátricas, Rio Grande do Sul. Rev Saude Publica, 2007;41(5):821-9. 10.1590/S0034-89102007000500017 17923904

[5] 5. Pereira PK, Santos SA, Lima LA, Legay LF, Santos JFC, Lovisi GM. Transtornos mentais e comportamentais no Sistema de Informações Hospitalares do SUS (SIH-SUS) no estado do Rio de Janeiro no período de 1999 a 2010. Cad Saude Colet. 2012;20(4):482-91. 10.1590/S1414-462X2012000400012

[6] 6. Szabzon F. Perfil das internações psiquiátricas em São Paulo: Um estudo exploratório [dissertação]. São Paulo: Universidade de São Paulo; 2013. 10.11606/D.6.2013.tde-20022014-095931

[7] 7. Coelho VAA, Volpe FM, Diniz SSL, Silva EM, Cunha CF. Alteração do perfil de atendimento dos hospitais psiquiátricos públicos de Belo Horizonte, Brasil, no contexto da reforma da assistência à saúde mental. Cienc Saude Colet. 2014;19(8):3605-16. 10.1590/1413-81232014198.11922013 25119099

[8] 8. Horta RL, Costa JSD, Balbinot AD, Watte G, Teixeira VA, Poletto S. Hospitalizações psiquiátricas no Rio Grande do Sul de 2000 a 2011. Rev Bras Epidemiol. 2015;18(4):918-29. 10.1590/1980-5497201500040019 26982305

[9] 9. Zurita RCM. Assistência psiquiátrica no estado do Paraná: análise das internações hospitalares no período de 2000 a 2013 [tese]. Maringá: Universidade Estadual de Maringá; 2015. Available from: http://repositorio.uem.br:8080/jspui/bitstream/1/1983/1/000222640.pdf

[10] 10. Mendes JDV. Evolução das causas de internação de saúde mental no SUS do Estado de São Paulo, 2000 a 2015. GAI Informa. 2016;8(51). Available from: http://portal.saude.sp.gov.br/resources/ses/perfil/profissional-da-saude/destaques//gais_51_abril_2016.pdf

[11] 11. Santos RS, Sena EP, Aguiar WM. Perfil de Internações psiquiátricas em unidade hospitalar de Salvador, Bahia. Rev Ciênc Méd Biol. 2017;16(3):374-9. 10.9771/cmbio.v16i3.24385

[12] 12. Lara APM, Volpe FM. Evolução do perfil das internações psiquiátricas pelo Sistema Único de Saúde em Minas Gerais, Brasil, 2001-2013. Cienc Saude Colet. 2019;24(2):659-68. 10.1590/1413-81232018242.14652017 30726398

[13] 13. Lima ALP, Santos L, Nery FS. Tendência temporal das internações psiquiátricas em Sergipe, entre 2008 a 2017. Cad Grad Ciênc Biol Saúde. 2019;5(3):179-92.

[14] 14. Guerra Junior AA, Pereira RG, Gurgel EI, Cherchiglia M, Dias LV, Ávila J et al. Building the national database of health centred on the individual: administrative and epidemiological record linkage-Brazil, 2000-2015. Int J Pop Data Sci. 2018;3(1):446. 10.23889/ijpds.v3i1.446 PMC8142958 34095519

[15] 15. Instituto Brasileiro de Geografia e Estatística. Censo demográfico 2010: características da população e dos domicílios. Rio de Janeiro: IBGE; 2011.

[16] 16. Instituto Brasileiro de Geografia e Estatística. Projeção da população das unidades da federação por sexo e grupos de idade: 2000-2030. Rio de Janeiro: IBGE; 2013.

[17] 17. Antunes JLF, Cardoso MRA. Uso da análise de séries temporais em estudos epidemiológicos. Epidemiol Serv Saúde. 2015;24(3):565-76. 10.5123/S1679-49742015000300024

[18] 18. Zanardo GLP, Silveira LHC, Rocha CMF, Rocha KB. Internações e reinternações psiquiátricas em um hospital geral de Porto Alegre: características sociodemográficas, clínicas e do uso da Rede de Atenção Psicossocial. Rev Bras Epidemiol. 2017;20(3):460-74. 10.1590/1980-5497201700030009 29160438

[19] 19. Mateus MD, Mari JJ, Delgado PG, Almeida-Filho N, Barrett T, Gerolin J et al. The mental health system in Brazil: policies and future challenges. Int J Ment Health Syst. 2008;2(1):12. 10.1186/1752-4458-2-12 PMC2553047 18775070

[20] 20. Mari JJ. Um balanço da reforma psiquiátrica brasileira. Cienc Saude Colet.2011;16(12):4593-6. 10.1590/S1413-81232011001300005 22124897

[21] 21. Global Health Observatory (GHO) data. Beds in general hospitals for mental health and beds in mental hospitals (per 100 000 population) [Internet]. Geneva: World Health Organization; 2019. Available from: http://www.who.int/gho/mental_health/care_delivery/beds_hospitals/en/

[22] 22. Balbinot AD, Horta RL, Costa JSD, Araújo RB, Poletto S, Teixeira MB. Hospitalizações por uso de drogas não se alteram com uma década de Reforma Psiquiátrica. Rev Saude Publica. 2016;50:26. 10.1590/S1518-8787.2016050006085 PMC4902101 27253902

[23] 23. Bastos FIPM, Vasconcellos MTLD, De Boni RB, Reis NBD, Coutinho CFDS. III Levantamento Nacional sobre o uso de drogas pela população brasileira. Rio de Janeiro: Fiocruz/ICICT; 2017. 528 p.

[24] 24. Bonadiman CSC, Passos VMA, Mooney M, Naghavi M, Melo APS. A carga dos transtornos mentais e decorrentes do uso de substâncias psicoativas no Brasil: Estudo de Carga Global de Doença, 1990 e 2015. Rev Bras Epidemiol. 2017;20(Supl 1):191-204. 10.1590/1980-5497201700050016 28658383

[25] 25. Zanardo GLP, Moro LM, Ferreira GS, Rocha KB. Factors associated with psychiatric readmissions: a systematic review. Paidéia (Ribeirão Preto). 2018;28:e2814. 10.1590/1982-4327e2814

[26] 26. Miliauskas CR, Faus DP, Junkes L, Rodrigues RB, Junger W. Associação entre internações psiquiátricas, cobertura de CAPS e atenção básica em regiões metropolitanas do RJ e SP, Brasil. Cienc Saude Colet. 2019;24(5):1935-44. 10.1590/1413-81232018245.18862017 31166526

[27] 27. Onocko-Campos RT, Amaral CEM, Saraceno B, Oliveira BDC, Treichel CAS, Delgado PGG. Atuação dos Centros de Atenção Psicossocial em quatro centros urbanos no Brasil. Rev Panam Salud Publica. 2018;42:e113. 10.26633/RPSP.2018.113 PMC6386118 31093141

[28] 28. Tomasi E, Facchini LA, Piccini RX, Thumé E, Silva RA, Gonçalves H et al. Efetividade dos centros de atenção psicossocial no cuidado a portadores de sofrimento psíquico em cidade de porte médio do Sul do Brasil: uma análise estratificada. Cad Saude Publica. 2010;26(4):807-15. 10.1590/S0102-311X2010000400022 20512220

[29] 29. Volpe FM, Braga IP, Silva EM. Community health services and risk of readmission in public psychiatric hospitals of Belo Horizonte, Brazil, 2005-2011. Trends Psychiatry Psychother. 2018;40(3):193-201. 10.1590/2237-6089-2017-0080 30304116

[30] 30. Rocha HA, Santos AF, Reis IA, Santos MAC, Cherchiglia ML. Saúde mental na atenção básica: uma avaliação por meio da Teoria da Resposta ao Item. Rev Saude Publica. 2018;52:17. 10.11606/S1518-8787.2018052000051

