# Supplementary material for: Psychiatric hospitalizations by the Unified Health System in Brazil between 2000 and 2014
Source: Rev Saude Publica. 2021 Apr 7;55:14. doi: 10.11606/s1518-8787.2021055002155 (PMC8030659; doi:10.11606/s1518-8787.2021055002155)
Supplement: Translation

# Translation: Internações psiquiátricas pelo Sistema Único de Saúde no Brasil ocorridas entre 2000 e 2014

**Authors:** Hugo André da Rocha, Ilka Afonso Reis, Marcos Antônio da Cunha Santos, Ana Paula Souto Melo, Mariangela Leal Cherchiglia

**Affiliations:** I Brasil Universidade Federal de Minas Gerais. Faculdade de Medicina. Programa de Pós-Graduação em Saúde Pública. Belo Horizonte, MG, Brasil; II Brasil Universidade Federal de Minas Gerais. Instituto de Ciências Exatas. Departamento de Estatística. Belo Horizonte, MG, Brasil; III Brasil Universidade Federal de São João Del Rei. Faculdade de Medicina. Divinópolis, MG, Brasil; IV Brasil Universidade Federal de Minas Gerais. Faculdade de Medicina. Departamento de Medicina Preventiva e Social. Belo Horizonte, MG, Brasil

**Keywords:** Transtornos Mentais, Hospital Dia, Hospitalização, Sistema Único de Saúde, Saúde Mental, Políticas Públicas de Saúde

## Abstract

**OBJETIVO:**

Caracterizar o perfil dos pacientes que foram internados por transtornos mentais e comportamentais pelo Sistema Único de Saúde (SUS) no Brasil entre 2000 e 2014, bem como verificar como aspectos da nova política de saúde mental influenciaram a taxa de pacientes internados no referido período.

**MÉTODOS:**

Estudo de coorte prospectiva não concorrente utilizando dados secundários de pacientes internados com diagnóstico primário de transtornos mentais e comportamentais entre 01/01/2000 e 31/12/2014. Foram selecionadas variáveis sociodemográficas, clínicas e de características do hospital, além disso, foram calculadas as taxas gerais de pacientes internados segundo motivo de internação, tipo de hospital, natureza jurídica e número de internações de cada paciente por ano. Foi testada a associação entre taxas de pacientes internados, número de leitos psiquiátricos por ano e número de Centros de Atenção Psicossocial por ano.

**RESULTADOS:**

Foram selecionados 1.549.298 pacientes dos quais os diagnósticos mais frequentes na primeira internação foram os transtornos devidos ao uso de substâncias psicoativas, seguidos por esquizofrenia e transtornos de humor. A mediana de internações por paciente foi de 1,9 e a de tempo de internação por paciente foi de 29 dias. A taxa geral de pacientes internados foi reduzida à quase metade no período. O número de leitos por ano apresentou associação positiva com as taxas de pacientes internados, e o número de CAPS por ano teve associação negativa com algumas taxas de pacientes internados.

**CONCLUSÃO:**

Verificou-se que, mesmo diante de um contexto de adversidades, a Política Nacional de Saúde Mental avançou em suas metas de reduzir progressivamente os leitos hospitalares e aumentar a oferta de serviços substitutivos de tal modo que ambas as estratégias foram associadas à redução das taxas de pacientes internados. Contudo, as mudanças foram percebidas com maior intensidade nos primeiros anos de implantação da política, tornando-se menos pujante nos últimos anos.

## INTRODUÇÃO

A Política Nacional de Saúde Mental (PNSM) passou, nos últimos anos, por significativas transições. A publicação da Lei 10.216, de 6 de abril de 2001, definiu o marco legal para a garantia dos direitos das pessoas portadoras de transtornos mentais, criando condições para que estas pudessem ser tratadas, preferencialmente, em serviços comunitários de saúde mental. A partir do momento em que a lei passou a ser vigente, a internação, em qualquer de suas modalidades, só seria indicada quando os recursos extra-hospitalares se mostrassem insuficientes e, para tanto, foi necessário investir na criação de serviços extra-hospitalares capazes de acolher a demanda e fornecer o devido tratamento^a^.

Os Centros de Atenção Psicossocial (CAPS) são serviços comunitários de saúde mental que atuam segundo a lógica de territórios. Entendidos como dispositivos estratégicos da política, são estruturas que atendem, prioritariamente, pessoas portadoras de transtornos mentais graves ou persistentes, bem como indivíduos com transtornos causados pelo uso de álcool e outras drogas. Estabelecidos em 2002, foram definidos conforme o porte populacional e o público-alvo (CAPS I, CAPS II, CAPS III, CAPS álcool e drogas (AD) e Infanto-juvenil (CAPS i))^b^. Após um período de grande expansão na implantação de novos serviços, verificou-se, nos últimos anos, desaceleração no processo. No período de 2002 a 2007 houve um crescimento médio anual de 25,9% de novos CAPS, já no período seguinte, de 2008 a 2014, o aumento médio anual foi de 9,8%^c^.

Simultaneamente à estruturação de novos dispositivos, ocorreu a redução progressiva de leitos hospitalares, a qual se deu de forma heterogênea nas diferentes regiões do país^1^. É importante citar o papel do Programa Nacional de Avaliação do Sistema Hospitalar/Psiquiatria (PNASH/Psiquiatria)^d^ e do Programa Anual de Reestruturação da Assistência Hospitalar Psiquiátrica no SUS (PRH)^e^ na reorganização da oferta de leitos hospitalares. Além disso, o lento avanço na implementação de serviços de funcionamento em tempo integral (CAPS III e CAPS AD III)^c^, capazes de garantir retaguarda aos episódios de crises, dificultou a redução das internações hospitalares^1^.

Estudos anteriores empreenderam o desafio de verificar alterações nas taxas de internação utilizando os dados disponibilizados pelo Datasus com abrangência municipal ou estadual^4^. Uma limitação imposta a estes estudos é a não identificação dos pacientes nos dados disponíveis, inviabilizando que indiquem o percurso de internações e análises individualizadas por paciente. Além disso, cabe destacar a importância de se investigar o papel das estratégias implementadas no âmbito da PNSM na redução das internações psiquiátricas.

Tendo em vista as considerações anteriores, apresentam-se como questões norteadoras do estudo: qual é o perfil dos pacientes internados por transtornos mentais e qual a influência das estratégias implementadas no comportamento das taxas de pacientes internados?

Nesse sentido, este estudo visa caracterizar o perfil dos pacientes que foram internados por transtornos mentais e/ou comportamentais pelo Sistema Único de Saúde no Brasil no período de 2000 a 2014, bem como verificar como aspectos da PNSM influenciaram a taxa de pacientes internados no referido período. Serão utilizados dados integrados por meio de relacionamento determinístico-probabilístico, no qual foi possível individualizar as internações de cada paciente

## MÉTODOS

O estudo utilizou uma coorte prospectiva não concorrente de dados secundários de pacientes selecionados na Base Nacional de Saúde. Trata-se de um banco de dados centrado no indivíduo, construído por meio de técnicas de pareamento determinístico-probabilístico de registros (*record linkage*) integrando os dados dos principais sistemas de informação do Sistema Único de Saúde: Sistema de Informações Ambulatoriais (SIA), Sistema de Informação Hospitalar (SIH) e Sistema de Informação de Mortalidade (SIM) de 2000 a 2015^14^.

Como critérios de inclusão, foram selecionados todos os pacientes que tiveram registro no SIH cujo diagnóstico primário referia-se a um código do Capítulo V na Classificação Estatística Internacional de Doenças e Problemas Relacionados à Saúde, 10ª Revisão – CID 10 Transtornos mentais e comportamentais (F00-F99), e cuja data de internação estivesse entre 01/01/2000 e 31/12/2014. Foram retirados os registros de pacientes com idade inferior a 18 anos na data da internação.

Para a descrição do perfil dos pacientes e das internações, foram selecionadas variáveis: I – sociodemográficas: sexo (feminino, masculino), idade (em anos) e região de residência (Norte, Nordeste, Sudeste, Sul, Centro-Oeste); II – clínicas: motivo de internação (diagnóstico primário), ocorrência de óbito no período (não, sim), ocorrência de óbito durante a internação psiquiátrica (não, sim), tempo de permanência em dias (somatório dos dias internados de cada paciente) e número de internações por paciente, III – características do estabelecimento: tipo de estabelecimento (hospital especializado, hospital geral, outros) e natureza jurídica do estabelecimento (público, privado, privado sem fins lucrativos).

As internações psiquiátricas são marcadas por longos períodos de duração, sendo indicado que as AIHs consecutivas mantenham o número da AIH inicial, mas muitas vezes são gerados novos registros, prática que acaba por mascarar a longa duração da internação psiquiátrica. Para amenizar esse efeito, conforme aplicado anteriormente por outros autores^12^, verificaram-se as AIHs de cada paciente, considerando-se como uma única internação quando a AIH subsequente tivesse ocorrido no mesmo hospital da AIH anterior e: a) a data de entrada da AIH subsequente era igual à data de saída da AIH anterior ou; b) a data de entrada da AIH subsequente apresentava um dia de diferença da data de saída da AIH anterior ou; c) a data de internação da AIH subsequente estava contida no período de internação da AIH anterior.

Para a análise descritiva das variáveis contínuas foi utilizada a mediana e o intervalo interquartil (IIQ), visto que a distribuição dos dados não foi simétrica. Os dados categóricos foram relatados como frequência e porcentagem.

Foram construídas séries temporais elencando os pacientes que foram internados em cada um dos anos do estudo (2000 a 2014), além disso, foram consideradas para o cálculo das taxas de pacientes internados a primeira internação do paciente que ocorreu em cada ano, não se considerando as possíveis reinternações. As internações que ultrapassavam o ano-calendário foram computadas no ano em que se iniciaram. As taxas de pacientes internados para cada ano foram calculadas considerando a seguinte equação:

$$\frac{\text{ número de pacientes internados no ano }}{\text{ população total no ano }(\geq 18\text{ anos })}\times 100.000\text{ habitantes }$$

Para os anos de 2000 e 2010, tomou-se como referência a população do censo^15^ e, para os demais, foram consideradas as projeções populacionais realizadas pelo Instituto Brasileiro de Geografia e Estatística^16^. As taxas de pacientes internados por ano foram padronizadas pelo método direto por sexo e idade, tendo como padrão a população brasileira de acordo com o censo de 2010^15^. Além da taxa geral de pacientes internados, foram calculadas as taxas específicas por: motivo da internação, tipo de hospital, natureza jurídica do hospital e número de internações de cada paciente por ano.

Foi utilizada a regressão em séries temporais para verificar se a variação identificada ao longo do período estava associada ao tempo. Neste modelo de regressão, a variável resposta é a própria série temporal e o tempo é a variável explicativa do modelo, de modo que a equação da regressão explica a variação da taxa de pacientes internados em função do tempo^17^. Cada uma das taxas de pacientes internados padronizada foi testada individualmente com a variável indicando o tempo em anos. Foram considerados estatisticamente significativos os modelos com p-valor < 0,05. O teste de Durbin-Watson foi utilizado para verificar a ocorrência de autocorrelação. As taxas de pacientes internados que apresentaram variação significativa foram selecionadas para a etapa seguinte de modelagem.

Considerando que, durante o período em estudo algumas estratégias, foram implementadas no âmbito da PNSM, foi verificado se duas dessas ações apresentavam associação com a alteração nas taxas de pacientes internados. As referidas estratégias analisadas foram a redução dos leitos psiquiátricos e a expansão dos CAPS. Foram consideradas, para essa análise, o número total de leitos disponível por ano e o número total de CAPS por ano (desconsiderando os CAPS i, voltados para atendimento de crianças e adolescentes, não incluídos neste estudo).

Todos os valores referentes às variáveis CAPS e número total de leitos disponíveis por ano acima foram consultados nos relatórios oficiais publicados pelo Ministério da Saúde^c,f^.

Para testar a associação entre as taxas de pacientes internados e as variáveis explicativas número de leitos e número de CAPS por ano, foi utilizado o modelo de mínimos quadrados generalizados (MQG) com processo autorregressivo de primeira ordem. Como as variáveis explicativas também se configuram como séries temporais, foi necessário utilizar um modelo de regressão capaz de incorporar a autocorrelação à estrutura de covariância dos erros. Para cada taxa de pacientes internados foram avaliados 2 modelos de regressão, um considerando os leitos por ano e outro com os CAPS por ano. Essas duas variáveis são altamente correlacionadas, tornando inviável qualquer modelo de regressão com ambas incluídas. A qualidade do ajuste dos modelos foi verificada pela análise gráfica dos resíduos e as análises estatísticas foram conduzidas no ambiente para programação estatística *R Project for Statistical Computing* (versão 3.6.1).

Este estudo é parte do projeto “Avaliação epidemiológica, econômica e de trajetórias assistenciais de procedimentos de alto custo no SUS: utilização de base de dados centrada no paciente a partir da integração de registros dos sistemas de informação em saúde”, aprovado pelo Comitê de Ética em Pesquisa da UFMG – (CAAE 44121315.2.0000.5149).

## RESULTADOS

Foram selecionados 1.549.298 pacientes, sendo a maioria do sexo masculino. A região do país com maior número de pacientes internados foi a Sudeste, e a região com menor número foi a Norte. Os grupos de causas de internação mais frequentes foram os transtornos devidos ao uso de substâncias psicoativas, seguidos por esquizofrenia e transtornos de humor. A ocorrência de óbito foi verificada para 12,2% dos pacientes, sendo que destes, 7,7% faleceram durante uma internação hospitalar psiquiátrica (Tabela 1).

**Tabela 1:** Características dos pacientes internados por transtornos mentais e/ou comportamentais pelo SUS e dos hospitais, Brasil, 2000–2014.

Pacientes	n	(%)
	1.549.298	(100)
Sexo		
Feminino	550.464	35,5
Masculino	998.834	64,5
Região		
Norte	33.651	2,2
Nordeste	303.886	19,6
Sudeste	673.688	43,5
Sul	403.229	26,0
Centro–Oeste	134.844	8,7
Causa de Internação por Grupo CID-10		
F10-F19 Transtornos mentais e comportamentais devidos ao uso de substância psicoativa	609.822	39,4
F20-F29 Esquizofrenia, transtornos esquizotípicos e transtornos delirantes	532.083	34,3
F30-F39 Transtornos do humor [afetivos]	238.164	15,4
F00-F09 Transtornos mentais orgânicos, inclusive os sintomáticos	88.569	5,7
F40-F99 Demais transtornos mentais	80.660	5,2
Óbito		
Não	1.35.528	87,8
Sim	189.770	12,2
Óbito durante internação psiquiátrica (n = 189.770)		
Não	175.207	92,3
Sim	14.563	7,7
Número de internações por paciente		
Uma	1.043.048	67,3
Duas	240.210	15,5
Três	162.212	10,5
Quatro ou mais	103.828	6,7
Tipo de hospital		
Especializado	1.020.276	65,9
Geral	461.943	29,8
Outros	35.197	2,2
N.A.*	31.882	2,1
Natureza jurídica do estabelecimento		
Privado sem fins lucrativos	551.526	35,6
Privado	511.378	33,0
Público	483.711	31,2
N.A.*	2.683	0,2

* Os estabelecimentos que tiveram registros apenas no período anterior a Agosto de 2005 não puderam ser classificados em virtude da limitação temporal dos dados do CNES.

A maioria dos pacientes teve uma única internação ao longo do período. As internações foram mais frequentes em hospital especializado e, em relação à natureza jurídica, a maior proporção de internações ocorreu em hospitais privados sem fins lucrativos.

A mediana de idade dos pacientes situou-se em 38 anos (intervalo interquartil [IIQ] 29:47). A mediana do número de internações de cada paciente foi de 1,9 vez durante todo o período estudado (IIQ 1–2), sendo a mediana do tempo de internação por paciente de 29 dias (IIQ 9–62). Foi verificado o total de 2.957.767 internações no período estudado (dados não apresentados em tabela).

A Figura apresenta as taxas de pacientes internados padronizadas por idade e sexo. A taxa geral de pacientes internados apresentou tendência de queda ao longo do período, passando de 188,5 no primeiro ano para 94,4 por 100 mil habitantes no último ano, configurando uma redução de 49,9%. Com relação à taxa de pacientes internados por tipo de hospital, observou-se a redução das internações em hospitais especializados (de 154,4 para 51,2) e o aumento simultâneo em hospitais gerais (de 24 para 46). Com relação à taxa por natureza do hospital destaca-se a redução de 84,32% de pacientes internados em hospitais privados, de 95,7 em 2000 para 15 por 100 mil habitantes em 2014.

**Figura:** Taxas geral e específicas de pacientes internados, Brasil, 2000–2014.

* Taxas de pacientes internados padronizadas por sexo e idade.

A taxa de pacientes internados específica por grupo de diagnóstico indica que houve redução de 66% nas taxas de pacientes internados por esquizofrenia (de 91,5 para 30,5), enquanto a taxa por uso de substâncias psicoativas apresentou redução de 29,8% (de 56,6 para 39,7). Sobre o número de internações por paciente, a taxa de pacientes submetidos a uma única internação por ano apresentou a mesma tendência de redução verificada para a taxa geral, de 137,3 em 2000 para 74 por 100 mil habitantes em 2014.

A regressão em séries temporais indicou haver uma variação significativa nas taxas de pacientes internados em função do tempo. Com exceção da taxa de pacientes internados em hospital geral, que apresentou tendência crescente, todas as demais taxas que apresentaram significância indicaram uma relação decrescente, ou seja, com tendência de redução ao longo do tempo. A taxa geral de pacientes internados apresentou redução de 5,72 pacientes internados por 100 mil habitantes a cada ano. Os coeficientes de cada um dos modelos podem ser verificados na Tabela 2.

**Tabela 2:** Coeficientes das regressões de séries temporais segundo taxa geral e específicas de pacientes internados, Brasil, 2000–2014.

Taxa de pacientes internados por ano padronizada	Intercepto (IC95%)^a^	Tempo (IC95%)^a^	R^2^ ajustado	p	Tendência
Taxa geral de pacientes internados	172,61 (149,82 a 195,39)	-5,72 (-8,22 a -3,21)	0,62	< 0,001	Redução
Taxa por grupo de causa					
F00-F09 transtornos orgânicos	14,22 (12,15 a 16,27)	-0,7 (-0,93 a -0,48)	0,76	< 0,001	Redução
F10-F19 uso de substâncias	53,28 (45,77 a 60,78)	-0,8 (-1,62 a 0,02)	0,19	0,06	^b^
F20-F29 esquizofrenia	82,87 (71,60 a 94,13)	-3,96 (-5,19 a -2,72)	0,77	< 0,001	Redução
F30-F39 transtornos de humor	20,57 (17,82 a 23,31)	0,03 (-0,27 a 0,32)	-0,07	0,85	^b^
F40 a F99 demais CIDs	11,17 (7,49 a 14,84)	-0,54 (-0,94 a -0,13)	0,34	< 0,05	Redução
Taxa por tipo de hospital					
Hospital especializado	144,82 (127,19 a 162,43)	-6,6 (-8,53 a -4,66)	0,79	< 0,001	Redução
Hospital geral	19,93 (15,86 a 24,01)	1,7 (1,25 a 2,14)	0,83	< 0,001	Aumento
Taxa por natureza jurídica do hospital					
Hospital Público	40,11 (33,69 a 46,53)	-0,32 (-1,02 a 0,38)	-0,003	0,35	^b^
Hospital Privado	91,14 (80,15 a 102,12)	-5,45 (-6,65 a -4,23)	0,87	< 0,001	Redução
Hospital Privado SFL	47,72 (40,54 a 54,90)	-0,09 (-0,88 a 0,69)	-0,07	0,80	^b^
Taxa por número de internações por ano de cada paciente					
Uma internação por ano	133,40 (119,60 a 147,19)	-4,16 (-5,67 a -2,64)	0,71	< 0,001	Redução
Duas internações por ano	24,93 (18,83 a 31,02)	-0,9 (-1,56 a -0,22)	0,34	< 0,05	Redução
Três internações por ano	8,84 (5,95 a 11,73)	-0,43 (-0,74 a -0,10)	0,35	< 0,05	Redução
Quatro ou mais internações por ano	6,86 (4,21 a 9,51)	-0,31 (-0,59 a -0,01)	0,23	< 0,05	Redução

^a^ IC 95%: intervalo de confiança de 95%.

^b^ não apresentou significância estatística.

Os modelos de mínimos quadrados generalizados indicaram associação da variável número de leitos por ano para quase todas as taxas de pacientes internados, com quase todos os modelos apresentando coeficientes positivos. Como os coeficientes apresentam valores baixos, é preciso adotar um fator de multiplicação para tornar mais simples a interpretação. Sendo assim, para a taxa de pacientes internados em hospital especializado, por exemplo, o aumento de 1000 leitos resultaria em aumento de 3 pacientes internados por 100 mil habitantes/ano. O número de CAPS por ano foi significativo em menos modelos, com coeficientes negativos. Considerando também a taxa de pacientes internados em hospital especializado, verificou-se que o aumento de 1000 unidades de CAPS acarretaria redução de 49 pacientes internados por 100 mil habitantes/ano. Todos os coeficientes e os respectivos intervalos de confiança (95%) são apresentados na Tabela 3.

**Tabela 3:** Coeficientes das regressões de mínimos quadrados generalizados segundo taxa geral e específicas de pacientes internados em função do número de CAPS por ano e do número de leitos psiquiátricos por ano, Brasil, 2000–2014.

Taxa de pacientes internados por ano padronizada	CAPS		Leitos	
	Coeficiente (IC95%)^a^	p	Coeficiente (IC95%)^a^	p
Taxa geral de pacientes internados	-0,043 (-0,09 a 0,004)	0,075	0,003 (0,001 a 0,004)^b^	< 0,001
Taxa por grupo de causa				
F00-F09 transtornos orgânicos	-0,005 (-0,008 a -0,002)	0,005	0,0003 (0,0002 a 0,0004)	< 0,001
F20-F29 esquizofrenia	-0,024 (-0,067 a 0,018)	0,242	0,002 (0,001 a 0,002)^b^	0,000
F40 a F99 demais CIDs	-0,004 (-0,017 a 0,008)	0,479	0,0003 (0,0000008 a 0,001)^b^	0,049
Taxa por tipo de hospital				
Hospital especializado	-0,049 (-0,083 a -0,016)	0,007	0,003 (0,002 a 0,003)^b^	< 0,001
Hospital geral	0,013 (0,009 a 0,017)	0,000	-0,001 (-0,001 a -0,00008)	0,025
Taxa por natureza jurídica do hospital				
Hospital Privado	-0,038 (-0,07 a -0,007)	0,020	0,002 (0,002 a 0,003)	< 0,001
Taxa por número de internações por ano de cada paciente				
Uma internação por ano	-0,03 (-0,053 a -0,008)	0,013	0,002 (0,001 a 0,003)^b^	< 0,001
Duas internações por ano	-0,006 (-0,015 a 0,002)	0,141	0,0004 (0,00005 a 0,0007)^b^	0,025
Quatro ou mais internações por ano	-0,003 (-0,011 a 0,005)	0,497	0,0004 (0,0001 a 0,0007)^b^	0,009

^a^ IC 95%: intervalo de confiança de 95%.

^b^ Intercepto não significativo (nível de significância 5%).

## DISCUSSÃO

Os resultados apresentados, utilizando uma coorte prospectiva não concorrente de abrangência nacional, indicam que houve mudanças nas internações por transtornos mentais no período de 2000 a 2014. Houve redução tanto em valores absolutos quanto em termos de taxas de pacientes internados, bem como mudanças no perfil dos serviços, com aumento das internações em hospitais gerais e redução em hospitais especializados. Cabe salientar que o presente estudo utiliza como principal indicador a taxa de pacientes internados (por 100 mil habitantes), que é diferente da taxa de internações (por 100 mil habitantes). Isso possibilitou avaliar a redução de números de indivíduos internados em cada ano.

Verificou-se que a maior parte da população analisada é de homens, o que corrobora outros estudos que também avaliaram internação de pacientes por transtornos mentais^5,7,11^. Um estudo realizado na região Sul do país identificou maior percentual de mulheres internadas em relação ao de homens, no entanto, é relevante considerar que tal estudo não incluiu pacientes internados por uso de álcool e outras drogas^18^. Com relação à idade, é possível perceber que se trata de pessoas em idade economicamente ativa, a distribuição etária indica que 75% dos pacientes tinha entre 18 e 47 anos de idade. Não é comum encontrar estudos com pacientes com média de idade avançada, concentrando-se a maior frequência de pacientes entre 30 e 49 anos de idade^5,7,11,18^.

Outros estudos que investigaram a tendência temporal das internações psiquiátricas também encontraram resultados que reforçam a hipótese de redução das taxas associadas ao tempo^9^. O estado do Rio Grande do Sul apresentou aumento das taxas de internações ao longo do tempo^8^.

As mudanças ocorridas nas taxas de pacientes internados por tipo de hospital e por natureza jurídica do hospital podem estar relacionadas às alterações promovidas na política hospitalar. A redução da taxa de pacientes internados em hospitais especializados foi concomitante ao aumento na taxa de pacientes internados em hospitais gerais, corroborando o que foi verificado em estudos anteriores^4^.

A internação psiquiátrica em hospitais gerais pode favorecer a redução do estigma, além de aumentar o acesso, melhorar o cuidado em relação à saúde física e possibilitar interconsultas entre as variadas especialidades médicas presentes neste tipo de hospital^4,19,20^. Dados do Observatório Global de Saúde indicam que o Brasil tem um déficit em relação ao número de leitos psiquiátricos em hospital geral. Enquanto o país possuía 0,6 leito/100.000 habitantes em 2016, países como Austrália, Espanha, Portugal e Itália possuíam 21,8, 14,3, 11,8 e 9,0 leitos/100.00 habitantes, respectivamente. Países vizinhos na América do Sul, como Uruguai, Chile e Argentina, contavam com 8,2, 5,2, e 2,7 leitos/100.000 habitantes, respectivamente^21^.

A grande redução na taxa de pacientes internados em hospitais privados sinaliza as mudanças na contratação de leitos psiquiátricos. Durante o período, muitos leitos e até mesmo hospitais inteiros foram descredenciados, conforme se verificou em estudo conduzido com dados do estado de Minas Gerais no período de 2001 a 2013^12^. Estudo publicado em 2007 indicou que no período de 1995 a 2005 houve redução de 41% no número de leitos psiquiátricos (5,4 a 3,2 por 10.000 habitantes)^2^. Entre 2008 e 2017, houve redução de 33,3% no número de leitos psiquiátricos no país^1^.

Notou-se uma redução importante na taxa de pacientes internados por esquizofrenia, a ponto de a taxa de pacientes internados devido ao uso de substâncias superá-la. Estudos anteriores verificaram aumento nas taxas de internação por transtornos devidos ao uso de substâncias, sendo que em alguns também foi verificada a redução na taxa de internações por esquizofrenia^8^.

A incipiente estruturação de serviços não hospitalares para atendimento de pacientes diagnosticados com transtornos por uso de substâncias psicoativas poderia explicar a crescente taxa de pacientes internados^7^. Os CAPS-ad III, que tratam desse perfil de pacientes em regime de funcionamento de 24 horas, ainda apresentavam baixa cobertura durante o período deste estudo, sendo que, em 2014, havia 69 serviços habilitados^c^em 51 municípios. Além disso, é preciso citar o aumento no consumo de drogas ilícitas pela população brasileira^23,24^, que a cada dia produz um contingente maior de pessoas que utiliza a internação hospitalar para tratar do uso abusivo^22^.

As taxas de pacientes internados uma ou duas vezes por ano tiveram menor redução do que as taxas de pacientes que internaram 3 e 4 ou mais vezes por ano. A alta frequência de internações para um mesmo paciente pode ser enquadrada no fenômeno conhecido como porta giratória (*revolving door*), no qual o paciente apresenta várias internações com intervalos curtos de alta entre elas. Uma revisão sistemática verificou que o diagnóstico de esquizofrenia apresentava maior risco para reinternação^25^. Nesse sentido, a redução da taxa entre pacientes que internaram mais vezes pode estar associada à grande contração verificada na taxa de pacientes internados por esquizofrenia.

A regressão em séries temporais indicou que a maioria das taxas de pacientes internados variou em função do tempo, em quase todos casos reduzindo-se a cada ano. Ao avaliar a associação dessas mudanças com as estratégias de redução dos leitos hospitalares e incremento do número de CAPS, notou-se que essas variáveis isoladas têm um efeito pequeno sobre a variação das taxas de pacientes internados. Aumentar o número de leitos implicaria o aumento de pacientes internados e, no sentido contrário, o crescimento do número de CAPS reduziria o número de pacientes internados.

Estudos anteriores investigaram a relação do CAPS com taxas de internação e reinternação, no entanto, os achados não são homogêneos. Estudo realizado em duas regiões metropolitanas do Sudeste indicou haver uma associação entre o aumento na cobertura dos serviços de CAPS com a redução das taxas de internação por transtornos mentais^26^. Em outro estudo, realizado nos municípios de Campinas, São Paulo, Porto Alegre e Fortaleza, verificou-se que menos de 10% dos usuários acompanhados nos CAPS necessitou de internação hospitalar em um período de 3 anos^27^. Em um estudo de coorte, foram observadas reduções significativas na ocorrência de crises, no uso de medicamentos e número de internações psiquiátricas entre os usuários com maior tempo de CAPS e em atendimento intensivo^28^. Contrastando estes resultados, um estudo conduzido nos 2 hospitais psiquiátricos públicos de Belo Horizonte – MG não identificou efeito protetor entre a cobertura de CAPS e a ocorrência de readmissão psiquiátrica^29^.

Pode-se presumir que a introdução dos CAPS tenha feito emergir uma demanda reprimida por serviços de saúde mental, que como dito anteriormente, não eram acessíveis e que à medida que vão se tornando disponíveis, vão inflacionando a demanda. Nesse sentido, cabe também citar que, nos últimos anos, houve grande aumento em termos de cobertura de equipes de atenção básica no país, que em algumas medidas incorporaram ações voltadas às pessoas portadoras de transtornos mentais. Muitas ações de saúde mental foram incorporadas pelas equipes de atenção básica, destacando-se a oferta de atendimentos em grupo e as ações educativas e de promoção da saúde^30^. Na medida em que há o aumento do acesso e consequentemente o de diagnósticos, aumenta também a pressão por serviços de média e alta complexidade para atender a esses casos.

A despeito da importância dos transtornos mentais na carga global de doenças, sendo responsáveis por 9,5% do total de anos de vida perdidos ajustados por incapacidade no Brasil em 2015^24^, o investimento necessário para lidar com essas condições não tem sido adequado. Um ponto fundamental da reforma, que seria a expansão de serviços extra-hospitalares para suprir a diminuição programada de leitos hospitalares, esbarra no limite orçamentário da política de saúde mental^19,20^.

Este trabalho apresenta algumas limitações, sendo uma delas o baixo número de variáveis sociodemográficas dos pacientes. Por tratar-se do uso de informações de bancos de dados oriundos de sistemas administrativos, muitas vezes informações como raça/cor, escolaridade e renda não se encontram presentes. Outro ponto verificado foi um volume menor de dados de pacientes internados entre os anos de 2004 e 2006, sendo esta uma limitação da Base Nacional de Saúde.

Este estudo permitiu ampliar o conhecimento sobre o perfil dos pacientes internados por transtornos mentais em nível nacional por dispor de uma base de dados abrangente e centrada no indivíduo. O protagonismo do hospital psiquiátrico na Política de Saúde Mental foi sendo diluído para outros serviços, mas sem romper com o modelo que durante muitos anos sustentou o vultuoso parque hospitalar que existia no país. Para tanto, seria necessário que os investimentos para a expansão dos serviços extra-hospitalares fossem ampliados, e não o contrário, como se observou recentemente. As novas e graves demandas em saúde mental solapam a capacidade de gestores e planejadores de saúde de fazer frente às necessidades para as quais o pior remédio é a austeridade.

## Footnotes

a Brasil. Lei nº 10.216, de 06 de abril de 2001. Dispõe sobre a proteção e os direitos das pessoas portadoras de transtornos mentais e redireciona o modelo assistencial em saúde mental. Diário Oficial da União. 9 abr. 2001.

b Brasil. Portaria/GM Nº 336 de 19 de fevereiro de 2002. Estabelece CAPS I, CAPS II, CAPS III, CAPS i II e CAPS ad II. Diário Oficial da União. 20 fev. 2020.

c Ministério da Saúde. Saúde Mental em Dados – 12. Saúde Mental em Dados. 2015;10(12).

d Brasil. Portaria nº 251, de 31 de janeiro de 2002. Estabelece diretrizes e normas para a assistência hospitalar em psiquiatria, reclassifica os hospitais psiquiátricos, define e estrutura a porta de entrada para as internações psiquiátricas na rede SUS e dá outras providências. Diário Oficial da União. 1 fev. 2002.

e Brasil. Portaria nº 52, de 20 de janeiro de 2004. Institui o Programa Anual de restruturação da Assistência Psiquiátrica Hospitalar no SUS. Diário Oficial da União. 20 jan. 2004.

f Ministério da Saúde. Saúde Mental em Dados – 8. Saúde Mental em Dados. 2011;6(8).
